# The Effectiveness of Alcohol Screening and Brief Intervention in Emergency Departments: A Multicentre Pragmatic Cluster Randomized Controlled Trial

**DOI:** 10.1371/journal.pone.0099463

**Published:** 2014-06-25

**Authors:** Colin Drummond, Paolo Deluca, Simon Coulton, Martin Bland, Paul Cassidy, Mike Crawford, Veronica Dale, Eilish Gilvarry, Christine Godfrey, Nick Heather, Ruth McGovern, Judy Myles, Dorothy Newbury-Birch, Adenekan Oyefeso, Steve Parrott, Robert Patton, Katherine Perryman, Tom Phillips, Jonathan Shepherd, Robin Touquet, Eileen Kaner

**Affiliations:** 1 Addictions Department, Institute of Psychiatry, King's College London, London, United Kingdom; 2 Centre for Health Service Studies, University of Kent, Canterbury, United Kingdom; 3 Department of Health Sciences, University of York, York, United Kingdom; 4 Teams Family Practice, Gateshead, United Kingdom; 5 Department of Psychological Medicine, Imperial College, London, United Kingdom; 6 Institute of Health and Society, Newcastle University, Newcastle, United Kingdom; 7 Northern Regional Drug and Alcohol Services, Newcastle, United Kingdom; 8 Faculty of Health and Life Sciences, Northumbria University, Newcastle, United Kingdom; 9 Division of Population Health Sciences and Education, St George's, University of London, London, United Kingdom; 10 Jeesal Cawston Park Hospital, Norfolk, United Kingdom; 11 Humber NHS Foundation Trust, Willerby, United Kingdom; 12 Violence Research Group, Cardiff University, Cardiff, United Kingdom; 13 Emergency Department, Kingston Hospital, Kingston upon Thames, London, United Kingdom; Centre for Addiction and Mental Health, Canada

## Abstract

**Background:**

Alcohol misuse is common in people attending emergency departments (EDs) and there is some evidence of efficacy of alcohol screening and brief interventions (SBI). This study investigated the effectiveness of SBI approaches of different intensities delivered by ED staff in nine typical EDs in England: the SIPS ED trial.

**Methods and Findings:**

Pragmatic multicentre cluster randomized controlled trial of SBI for hazardous and harmful drinkers presenting to ED. Nine EDs were randomized to three conditions: a patient information leaflet (PIL), 5 minutes of brief advice (BA), and referral to an alcohol health worker who provided 20 minutes of brief lifestyle counseling (BLC). The primary outcome measure was the Alcohol Use Disorders Identification Test (AUDIT) status at 6 months. Of 5899 patients aged 18 or more presenting to EDs, 3737 (63·3%) were eligible to participate and 1497 (40·1%) screened positive for hazardous or harmful drinking, of whom 1204 (80·4%) gave consent to participate in the trial. Follow up rates were 72% (n = 863) at six, and 67% (n = 810) at 12 months. There was no evidence of any differences between intervention conditions for AUDIT status or any other outcome measures at months 6 or 12 in an intention to treat analysis. At month 6, compared to the PIL group, the odds ratio of being AUDIT negative for brief advice was 1·103 (95% CI 0·328 to 3·715). The odds ratio comparing BLC to PIL was 1·247 (95% CI 0·315 to 4·939). A per protocol analysis confirmed these findings.

**Conclusions:**

SBI is difficult to implement in typical EDs. The results do not support widespread implementation of alcohol SBI in ED beyond screening followed by simple clinical feedback and alcohol information, which is likely to be easier and less expensive to implement than more complex interventions.

**Trial Registration:**

Current Controlled Trials ISRCTN 93681536

## Introduction

Alcohol makes a significant contribution to the global burden of disease, injury and economic cost [1]. Over 20 million people are treated in emergency departments (ED) in England each year of which 35% of attendances are alcohol related, rising to 40% of attendances at weekends and up to 70% at peak times [2], [3]. Such presentations offer the opportunity for early identification and intervention to reduce hazardous and harmful drinking [4], [5].

There is a substantial evidence base for the efficacy of opportunistic screening and brief interventions (SBI) to reduce hazardous and harmful drinking in primary health care [6]. The evidence in ED is currently inconclusive. We conducted a rapid systematic review of SBI trials in ED (S1 Text) excluding studies conducted in adolescents only, trauma centres, and ED studies only including injured patients, to allow comparability to the current study. We identified six randomized controlled trials and two systematic reviews [6]–[13]. All identified trials were single site studies, four of which were in university teaching hospitals and mostly delivered by specialist staff employed by the study team rather than ED staff. Four of the trials assessed efficacy rather than effectiveness. Only one trial had a significant effect of intervention on alcohol consumption at 12 months [12]. A further trial had a significant effect on consumption at 6 but not 12 months, and reduced re-attendances at 12 months [8]. One trial found that patients receiving no intervention fared significantly better than those randomized to motivational interviewing [9].

ED is a busy environment with high patient and junior doctor turnover making SBI challenging to implement: one UK trial was abandoned due to low uptake of screening and intervention, whilst in another trial, though successful, initial data collection by ED staff was of necessity limited [8], [14]. So while alcohol SBI in ED shows some promise in single site trials, its effectiveness in the typical ED setting was unknown. Further the optimal intensity of SBI was unknown [6], [15]. The current study (SIPS ED trial) is the first pragmatic multicentre RCT of SBI in typical EDs. It included a larger sample size than previous trials, and cluster randomization to reduce contamination between intervention conditions. It was commissioned by the UK Department of Health as a facet of the Alcohol Harm Reduction Strategy for England [16]. The wider SIPS research programme included two related cluster RCTs in primary health care and criminal justice agencies and a health economic evaluation which are reported separately [17]–[19].

## Methods

### Ethics statement

The study received ethical approval from the London Research Ethics Committee (reference number: 07/MRE02/06).

### Trial design and participants

The trial methodology is described here in brief. The protocol for this trial and CONSORT checklist are available as Checklist S1 and Protocol S1. We conducted a pragmatic factorial cluster randomized trial of alcohol SBI in nine EDs across three English regions (North East, South East, London). Participating EDs were selected on the basis of having no current routine alcohol SBI programme, representing a broad cross section of EDs including rural, suburban, urban and metropolitan catchment areas with wide ethnic and sociocultural diversity, located in both teaching hospitals and typical district general hospitals.

Our aim was that all patients aged 18 years or older who attended the participating EDs and otherwise met the inclusion criteria would be screened by ED staff using one of three short validated alcohol screening tools: the modified Single Alcohol Screening Question (M-SASQ), FAST Alcohol Screening Test, or a modified version of Paddington Alcohol Test (SIPS-PAT) [19]. EDs were randomly assigned to one of the three screening approaches. Inclusion criteria were age > = 18 and screening positive on an alcohol screening test, being sufficiently alert and orientated to provide informed consent, living within the catchment area of the ED, and being able to speak read or write English sufficiently well to complete study questionnaires. Exclusion criteria were patients who were age <18, already seeking alcohol treatment, participating in another study of alcohol interventions, severe injury, or suffering from a serious mental health problem, or grossly intoxicated, or being of no fixed abode.

### Procedures

Those patients screening positive on the relevant alcohol tool were invited by ED or research staff to provide informed written consent to participate in the trial. The aim was to have all eligibility, screening, consent and baseline data collection carried out by ED staff. Furthermore ED staff were trained to deliver the interventions according to the condition they were allocated to. However, due to a low level of ED staff participation, this was carried out by the research team in six of nine EDs. The baseline assessment included demographic data, an extended item version of the Alcohol Use Disorders Identification Test, and a modified Readiness Ruler [19]. Participants were sent a voucher with a value of £10 following completion of the baseline interview.

We compared three different alcohol interventions of different intensity and complexity, with three EDs randomized to each condition, creating nine clusters in total. Participants in the minimal intervention control group were provided with simple clinical feedback using a standard script that their test result indicated they were drinking above the government's “safe” drinking levels, and were given a Patient Information Leaflet (PIL): the Department of Health's “*Drinking and You: How Much is Too Much?*” leaflet, including information on local alcohol services where further help could be sought by the patient themselves [19].

The intermediate intervention was the provision of 5 minutes of brief advice (BA) about drinking using the SIPS brief advice tool (*Brief Advice About Alcohol Risk*) developed for the trial and was based on the *How much is too much?* intervention pack developed as part of the UK version of the WHO collaborative Drink-Less Brief Intervention programme by Northumbria and Newcastle Universities [19]. Following brief advice the PIL was delivered in the same manner as in the minimal intervention group.

The more intensive intervention was Brief Lifestyle Counseling (BLC) delivered by SIPS employed Alcohol Health Workers (AHW) with specialist training and experience in alcohol motivational interventions. This was a 20 min lifestyle counseling alcohol intervention based on the *How much is too much?* intervention pack originally developed by Northumbria and Newcastle Universities, informed by the work of Rollnick and colleagues [19]–[20]. The procedure was that ED staff would first deliver the BA and PIL as above and then refer the patient to the SIPS AHW with an appointment the following day or as soon as possible thereafter.

At the intake point participants were invited to give their preference of follow up method - either by telephone, email or postal questionnaire. The protocol allowed for changing from the preferred method of follow up to the other methods if this proved unsuccessful. Most opted for telephone follow up and many who preferred other methods were successfully followed up by telephone. Telephone follow up was conducted by researchers who were blind to the participants allocated intervention condition.

The primary outcome measure was the AUDIT status (score of <8 versus > = 8) on the extended item AUDIT questionnaire at 6 months post consent.

Secondary outcome measures were average number of drinks per day using the quantity-frequency questions of the extended AUDIT, alcohol related problems using the Alcohol Problems Questionnaire (APQ), readiness to change using a modified Readiness Ruler, all of which were measured at 6 and 12 months, and patient satisfaction using a modified version of the Patient Satisfaction Questionnaire measured at 12 months only [19]. We have noted that there are some discrepancies in the definition of our primary outcome in previous published documents of our study: Primary outcome in trial registration: “Alcohol Use Disorders Identification Test (AUDIT) at baseline and 6 months”.

Primary outcome in protocol paper: “the score on the AUDIT screen at 12 months post-consent”.

Primary outcome in this manuscript: “the AUDIT status (score of <8 versus > = 8) on the extended item AUDIT questionnaire at 6 months post consent”.

To clarify this issue the primary outcome as stated in the registration is the primary outcome tool and this is operationalised as AUDIT status (score of <8 versus > = 8) at 6 months, this is the same as in this manuscript. In the BMC protocol paper we use the term score on the AUDIT screen which in effect is an alternative term for the AUDIT status. We can see that an error has occurred in the BMC protocol paper as we have used a 12 month time-point for the primary outcome rather than 6, as stated in the registration and submitted paper and the outcome the original study was powered to assess. We will contact the relevant editor at BMC to have this amended.

### Randomization and masking

Randomization was conducted using a secure remote randomization service. Nine allocations were generated for each of the possible factorial combinations of screening method (SIPS-PAT, FAST, M-SASQ) and intervention condition (PIL, BA, BLC). EDs and allocations were randomly sampled without replacement and paired to generate allocation groups.

Participants were informed that they would be taking part in a study comparing different types of alcohol intervention taking place in different EDs. However they were only informed of the intervention taking place in their ED. Staff in each ED were informed of the design of the study and provided with a basic description of the different interventions being compared in the trial. However local ED staff were only trained to administer the intervention appropriate to their randomized ED allocation. The research team and SIPS employed AHWs were aware of the study design and were trained in all intervention methods. Researchers conducting 6 and 12 month follow up were blinded to the participants' allocated treatment condition and efforts were made to prevent participants from inadvertently revealing the intervention they received.

### Sample size and data analysis

Recent meta-analysis suggests that the difference between brief intervention and control in alcohol consumption is 13%; 5% reduction in the control group and 18% in the brief intervention group [21]. We employed an established formula in our sample size calculation [22] and in order to detect this difference at the 5% significance level with 80% power, for a two-sided test, requires 109 patients in each of the three groups, a total of 327. Assuming a loss to follow up of 25% inflates the sample required to 131 in each group, a total of 393 patients. The proposed study involves a cluster design and requires a statistical adjustment to account for any potential cluster effect. The literature and our previous experience of trials in primary care suggest that is appropriate. Assuming an intra-class correlation coefficient of 0·04, a cluster size of the order 44 patients, this inflates the sample size calculation by a factor of 2·7 requiring a total of 1179 patients, 393 in each group, with an expectation that at least 882 will be followed up at 6 months and 12 months.

The primary analysis was by intention to treat, whereby participants are analysed as members of their allocated group irrespective of the treatment received to provide a pragmatic estimate of effectiveness, using a weighted linear regression model. The data were summarised for each of the nine clusters, summing the total number of AUDIT positive and AUDIT negative patients. The average baseline AUDIT score for each cluster was also calculated. For each cluster the odds of being AUDIT negative (low alcohol risk) were computed. For the primary analysis, the log odds of being AUDIT negative at 6 months were used as the dependent variable. In order to adjust for baseline differences in clusters, baseline AUDIT score was included in the model in addition to intervention and screening method. The analysis was weighted for the number of patients in each cluster responding at month six. The results were transformed and then presented as odds ratios. We explored the impact of missing data on the primary outcome by conducting multiple imputations and assessing the impact of missing data using sensitivity analysis. Weighted analyses were conducted for all other binary measures. Continuous variables were analysed using the mean score from each cluster which was then used in a weighted linear regression model. A per protocol analysis was conducted for the primary outcome including only those who received their allocated intervention. For the analysis of readiness to change ruler, the four categories were collapsed to form two categories, effectively those who were thinking of changing or had actually changed drinking and those who had not. All analyses were performed in STATA version 10.

## Results

### Implementation, recruitment and follow up

Screening and interventions were carried out by ED staff in three of 9 EDs and of necessity, due to low ED staff participation, by SIPS employed staff in the remaining six: two of three in the PIL condition, one of three in the BA condition and all in the BLC condition.

**Figure 1:**
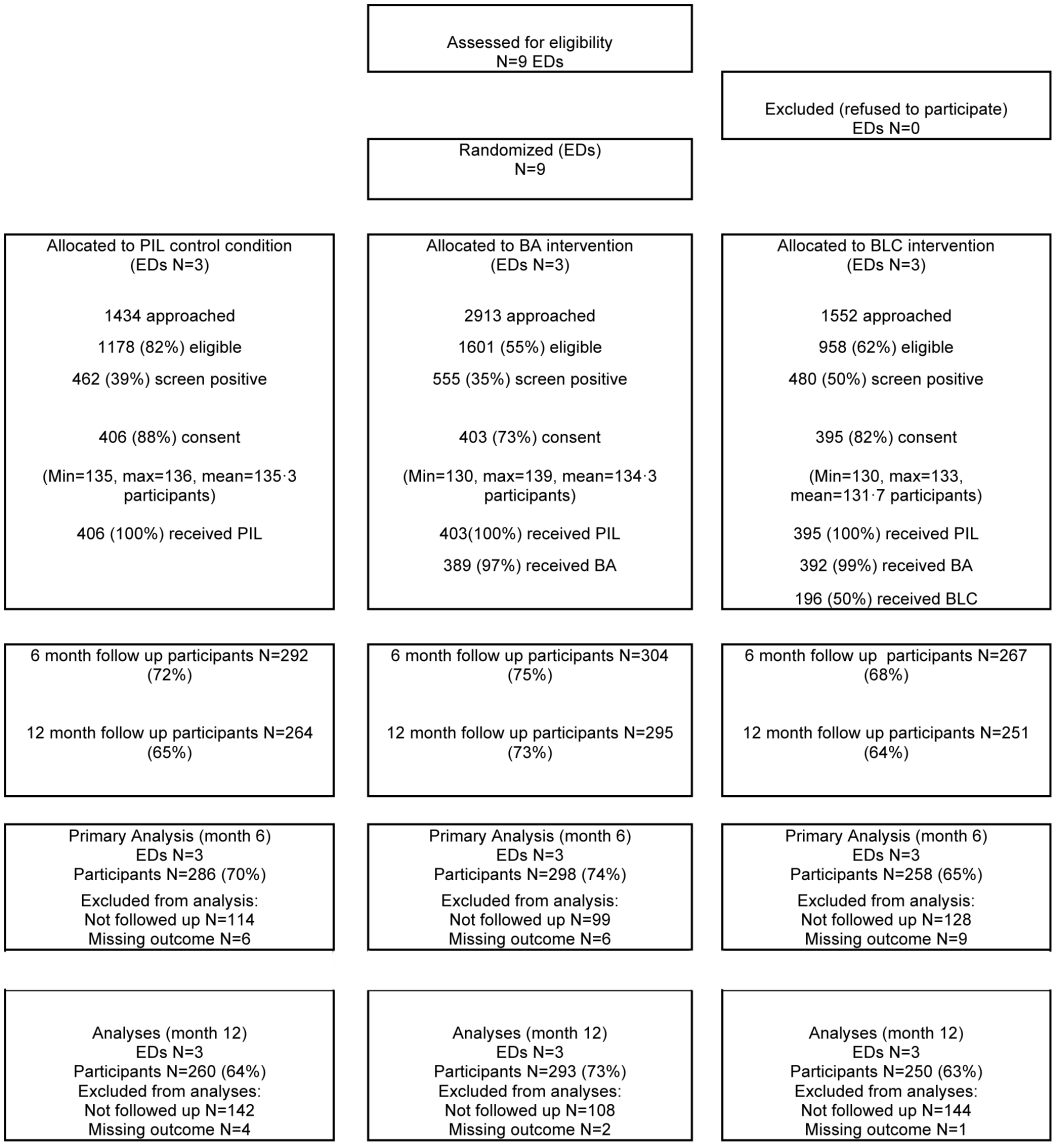
Consort Diagram.

Recruitment started in March 2008 and finished in April 2009, and 12 month follow up was completed in May 2010. The Consort statement for the study is shown in Figure 1. A total of 5899 potential participants were assessed for eligibility for the trial, of whom 3737 (63·3%) were eligible to participate. Reasons for ineligibility are shown in Table 1. The commonest reasons for ineligibility were not being alert and orientated (26·1%), unable to speak English (21·1%), and not providing verbal consent to be screened (16·1%). Of those eligible 1497 (40·1%) screened positive for alcohol misuse, of whom 1204 (80·4%) gave consent to participate in the trial, with similar numbers across intervention groups (PIL n = 406; BA n = 403; BLC n = 395).

**Table 1:** Reasons for ineligibility.

Reason	n	% of ineligible
Not alert and orientated	564	26·1
Does not speak English	457	21·1
Refused verbal consent to screen	349	16·1
Not resident within 20 miles of ED	260	12·0
Serious mental health problem	120	5·6
No fixed abode	113	5·2
Gross alcohol intoxication	106	4·9
Already seeking help for an alcohol problem	90	4·2
Severely injured	79	3·7
Already participating in a research project	24	1·1
**Totals**	**2162**	**100**

All patients in EDs allocated to PIL condition received the intervention. In the BA condition all patients received the PIL and 97% received BA. In the BLC condition, all patients received PIL, 99% received BA, and 50% received BLC.

Overall the follow-up rate at 6 months was 72% (n = 863), and was higher for those in the BA group (75%) than those allocated to PIL (72%) and BLC (68%). At 12 months the follow up rate was 67% (n = 810), higher in the BA group (73%) than either the PIL group (65%) or the BLC group (64%). These differences in follow up rate were not statistically significant.

### Sample characteristics

The characteristics of the sample are shown in Table 2. Overall the mean age of those consenting to the study was 34·6 years and was similar across screening and intervention groups. The majority of the sample was male (65%). Overall 88% of the sample classified their ethnicity as white, with more white participants in the PIL group (93%) than either the BA or BLC groups (both 85%). The proportion of single people was 54%, with more in the BLC group (62%) than either the PIL (53%) or BA (48%) groups. Almost 65% of the sample continued with education after the age of 16 years. A smaller proportion of participants in the PIL group (59%) had continued in education than either the BA (66%) or BLC (69%) groups. Overall 39% of participants had a degree or equivalent. A higher proportion of those in the BLC group (48%) had a degree than either the BA (37%) or PIL (33%) groups. In excess of 45% of participants were current smokers.

**Table 2:** Demographic and baseline measures by intervention allocation.

		Minimal (N = 406)		Brief Advice (N = 403)		Brief Lifestyle Counseling (N = 395)		Total (N = 1204)	
		**N**	**Mean (SD)**	**N**	**Mean (SD)**	**N**	**Mean (SD)**	**N**	**Mean (SD)**
Age in years		402	34·1 (12·6)	402	35·2 (14·3)	394	34·5 (13·3)	1198	34·6 (13·4)
Average drinks per day		401	2·4 (2·1)	400	2·1 (2·0)	389	2·2 (2·1)	1190	2·3 (2·1)
		**N**	**%**	**N**	**%**	**N**	**%**	**N**	**%**
Gender	Male	272/404	67·3%	270/403	67·0%	242/395	61·3%	784/1202	65·2%
Ethnicity	White	372/401	92·8%	343/402	85·3%	334/395	84·6%	1049/1198	87·6%
Marital Status	Single	215/403	53·3%	192/402	47·8%	245/393	62·3%	652/1198	54·4%
Education after 16 years	Yes	240/404	59·4%	267/402	66·4%	273/395	69·1%	780/1201	64·9%
Possess degree or equivalent	Yes	130/398	32·7%	148/400	37·0%	182/382	47·6%	460/1180	39·0%
Smoke tobacco	Current smoker	198/404	49·0%	172/401	42·9%	172/395	43·5%	542/1200	45·2%
Readiness Ruler	Never think about drinking less	149/401	37·2%	141/397	35·5%	143/390	36·7%	433/1188	36·4%
	Sometimes think about drinking less	135/401	33·7%	145/397	36·5%	142/390	36·4%	422/1188	35·5%
	I have decided to drink less	41/401	10·2%	44/397	11·1%	43/390	11·0%	128/1188	10·8%
	Already trying to cut down	76/401	19·0%	67/397	16·9%	62/390	15·9%	205/1188	17·3%

The mean AUDIT score at baseline was 12·4 (SD 6·9)(Table 3). The PIL group had a higher baseline AUDIT score 13·3 (SD 6·9) than either BA 12·2 (SD 7·0) or BLC 11·7 (SD 6·6). Overall 22·1% were AUDIT negative (PIL 14·9%, BA 24·5%, BLC 27·2%).

**Table 3:** Baseline AUDIT score.

		Minimal		Brief Advice		Brief Lifestyle Counseling		Total		ICC
		**N**	**%**	**N**	**%**	**N**	**%**	**N**	**%**	
AUDIT Status	NEGATIVE (<8)	59	14·9%	96	24·5%	103	27·2%	258	22·1%	0·04 (0·02)
	POSITIVE (> = 8)	336	85·1%	296	75·5%	276	72·8%	908	77·9%	
AUDIT Category	NEGATIVE (<8)	59	14·9%	96	24·5%	103	27·2%	258	22·1%	0·03 (0·02)
	HAZARDOUS (8–15)	228	57·7%	208	53·1%	199	52·5%	635	54·5%	
	HARMFUL (> = 16)	108	27·3%	88	22·4%	77	20·3%	273	23·4%	
AUDIT score		**N**	**Mean (SD)**	**N**	**Mean (SD)**	**N**	**Mean (SD)**	**N**	**Mean (SD)**	
		395	13·3 (6·9)	392	12·2 (7·0)	379	11·7 (6.6)	1166	12·4 (6·9)	0·02 (0·01)

### Clinical effectiveness

In the primary analysis, the proportion of AUDIT negative was 27·6% in PIL, 34·5% in BA and 39·5% BLC at 6 months (Table 4). The odds ratio comparing BA to PIL was 1·103 (95% CI 0·328 to 3·715) and comparing BLC to PIL was 0·690 (95% 0·315 to 4·939). These were not statistically significant differences. Multiple imputation of missing data found no significant differences so the outcome reported is based on observed values at 6 months. Differences in AUDIT status at 12 months also were not significantly different between intervention groups.

**Table 4:** Outcome measures at 6 and 12 months.

	Minimal Intervention	Brief Advice	Brief Lifestyle Counseling	Brief Advice/Minimal*		Brief Lifestyle Counseling/Minimal*		ICC (se)
				OR	P-value	OR	P-value	
	N (%)	N (%)	N (%)	(95% CI)		(95% CI)		
**Intention to treat analysis**								
AUDIT Negative a - Month 6	79/286 (27·6)	103/298 (34·5)	102/258 (39·5)	1·1	0·81	1·2	0·65	0·02 (0·02)
				(0·3 to 3·7)		(0·3 to 4·9)		
AUDIT Negative a - Month 12	91/260 (35·0)	123/293 (42·0)	108/250 (43·2)	1·0	0·88	0·9	0·69	0·02 (0·01)
				(0·4 to 2·5)		(0·3 to 2·6)		
RCQ – Changed – Month 6^b^	121/287 (42·2)	105/297 (35·4)	94/261 (36·0)	0·8	0·23	0·8	0·36	0·01 (0·01)
				(0·5 to 1·3)		(0·5 to 1·4)		
RCQ – Changed – Month 12^b^	112/253 (44·3)	106/286 (37·1)	82/244 (33·6)	0·8	0·18	0.647	0·08	0·01 (0·01)
				(0·5 to 1·3)		(0·4 to 1·1)		

	N	N	N	Mean Difference	P-value	Mean Difference*	P-value	
	Mean (SE)	Mean (SE)	Mean (SE)	(95% CI)		(95% CI)		
AUDIT Score – Month 6 a	286	298	258	−0·4	0·72	−0·4	0·77	0·02 (0·02)
	11·2 (0·7)	10·8 (0·6)	10·8 (0·7)	(−3·5 to 2·7)		(−3·9 to 3·2)		
AUDIT Score – Month 12 a	260	293	250	−0·5	0·70	−0·5	0·71	0·02 (0·01)
	10·3 (0·9)	9·8 (0·7)	9·8 (0·8)	(−4·1 to 3·1)		(−4·7 to 3·6)		
APQ Score – Month 6^a^	286	298	258	0·2	0·75	0·4	0·47	0·01 (0·01)
	2.7 (0.3)	2.9 (0.3)	3.2 (0.3)	(−1·3 to 1·6)		(−1·2 to 2·1)		
APQ Score – Month 12^a^	263	295	251	−0·5	0·27	−0·1	0·89	0·01 (0·01)
	2.7 (0.3)	2.2 (0.2)	2.6 (0.2)	(−1·6 to 0·6)		(−1·3 to 1·2)		
ADD – Month 6^c^^,^ +	291	300	264	0.0	0.95	−0.2	0.33	0.01 (0.01)
	2.4 (0.13)	2.1 (0.11)	2.0(0.12)	(−0.7 to 0.7)		(−0.8 to 0.4)		
ADD – Month 12^c^^,^ +	263	294	251	0.1	0.78	−0.2	0.38	0.02 (0.02)
	2.2 (0.13)	1.9 (0.11)	1.8 (0.12)	(−0.6 to 0.7)		(−0.8 to 0.4)		

a adjusted for mean baseline AUDIT score and screening instrument.

b adjusted for baseline log odds of being in change group and screening instrument.

c adjusted for baseline ADD and screening instrument.

*estimates of differences are produced from weighted regression models.

+ ADD – average drinks per day.

Similarly no significant differences were found between intervention conditions for average drinks per day, AUDIT score, APQ score or readiness to change, at 6 or 12 months. All results are presented in Tables 4 and 5.

**Table 5:** Outcome measures at 6 and 12 months.

	Minimal Intervention N (%)	Brief Advice N (%)	Brief Lifestyle Counseling N (%)	Brief Advice/Minimal*		Brief Lifestyle Counseling/Minimal*		ICC (se)
	N	N	N	Mean Difference	P-value	Mean Difference*	P-value	
	Mean (SE)	Mean (SE)	Mean (SE)	(95% CI)		(95% CI)		
General Satisfaction – Month 12^d^	240	268	234	−0.06	0.31	−0.12	0.08	0.01 (0.01)
	4.01 (0.04)	3.95(0.04)	3.90 (0.04)	(−0.19 to 0.07)		(−0.26 to 0.02)		
Communication – Month 12^d^	241	269	231	−0.08	0.25	−0.04	0.52	0.01 (0.01)
	4.14 (0.04)	4.06 (0.04)	4.09 (0.04)	(−0.25 to 0.09)		(−0.22 to 0.13)		
Interpersonal manner – Month 12^d^	240	269	233	0.01	0.90	−0.02	0.81	0.01 (0.01)
	4.04 (0.04)	4.05 (0.03)	4.02 (0.04)	(−0.21 to 0.23)		(−0.25 to 0.21)		

Combined analysis	Minimal N (%)	Combined Treatment N (%)			Combined/Minimal		
					OR	P-value	
AUDIT Negative - Month 6^a^	79/286 (27·6)	205/556 (36·9)			1·1	0·69	0·02 (0·02)
					(0·5 to 2·8)		

Per protocol analysis								
AUDIT Negative - Month 6^a^	79/286 (27·6)	99/289 (34·3)	49/133 (36·8)	1·1	0·78	1·5	0·23	0·03 (0·02)
				(0·3 to 3·5)		(0·6 to 3·9)		

a adjusted for mean baseline AUDIT score and screening instrument.

d adjusted for screening instrument.

*estimates of differences are produced from weighted regression models.

A per protocol analysis based on interventions actually received by patients, and an analysis comparing both BA and BLC in a combined group to PIL also failed to find any significant differences between the intervention groups at 6 months (Tables 4 and 5).

## Discussion

This study has important implications for alcohol screening and brief intervention (SBI) in ED. The original design of the study was for the SBI to be delivered by ED staff, apart from the BLC intervention. The latter required ED staff to refer patients to an alcohol health worker (AHW) for a subsequent consultation usually a few days after initial ED attendance, this being comparable to the St Mary's model [8]. However due to low participation of ED staff the study team had to deliver the SBI in six out of nine EDs. The implication is that, although there is some enthusiasm amongst ED staff to carry out alcohol interventions, it is likely to be difficult to implement SBI in the typical ED setting without significant external support from specialist alcohol staff.

The results showed that in a large pragmatic multicentre RCT, there was no significant difference in outcome between the three intervention conditions either in intention to treat or per protocol analyses on any of the outcome measures. These results are largely consistent with the systematic review, with the exception of two out of six single site efficacy studies conducted in university teaching hospitals which found significant effects of more intensive intervention [8]–[12]. This suggests that beyond the provision of simple clinical feedback and an alcohol information leaflet, more intensive interventions do not add significant clinical benefit.

Only 50% of patients referred for BLC intervention actually received it. Although this is a higher attendance rate than the previous UK ED trial (29·3%) and an Australian trial (10%), it suggests non-attendance at subsequent outpatient appointments following ED attendance may limit the effectiveness of BLC in typical practice [8], [9]. Also previous research has shown that longer delay in receiving an appointment with an AHW results in greater attrition [23].

The strengths of this study include the fact that it is the first large pragmatic multicentre RCT of effectiveness of SBI in typical EDs, and rates of eligibility and consent were higher than in previous SBI studies, which adds weight to the generalisability of the research. Further we did not exclude patients with alcohol dependence as some previous studies have, since there is some evidence to suggest more dependent drinkers might benefit more from SBI in ED than hazardous drinkers [24], [25]. Cluster randomization reduced the potential for contamination between interventions being delivered within a single clinical site with the potential for subversion of the protocol.

Weaknesses of the study include that we achieved a lower follow-up rate than planned (70% at 6 months and 67% at 12 months, compared to 75% planned) which will have reduced the statistical power, although these follow up rates are comparable with previous trials in ED. As this was a pragmatic effectiveness trial there was more limited measurement of the fidelity of the interventions in order to more closely represent typical practice. It is therefore possible that the lack of differences between intervention groups may have been due to unsuccessful implementation of the clinical protocols. Further, in six out of nine EDs the clinical protocols were implemented by study staff so the intervention being evaluated differed from the protocol. However as this was a pragmatic trial, the introduction of an AHW to deliver SBI reflects what is likely to have occurred with implementation in typical practice.

The study did not include a ‘no intervention’ group as a comparator with more intensive interventions. It is therefore not possible to conclude that the reductions in hazardous and harmful drinking in all three conditions can be attributed to the interventions rather than ‘assessment reactivity’ or regression to the mean effects.^26^ However three ED trials which have included patients who were only screened and followed up did not show differences in outcome with patients who were assessed and enrolled in the trial interventions [11], [12], [27].

Viewed in the context of our systematic review these findings add to a growing body of evidence that suggest ED is a less useful setting in which to implement alcohol SBI than in primary health care where the evidence is considerably stronger [6]. This might be related to several differences between the settings. Primary care staff are likely to have a more effective and ongoing therapeutic relationship with patients, which may provide a better context for SBI compared to the transient nature of ED attendance. Primary care has a more established role in providing preventive lifestyle interventions including diet and smoking, which may increase the legitimacy of alcohol SBI for both practitioners and patients. Patients often present to ED at a point of crisis which may be accompanied by distress and/or alcohol intoxication, and this might limit patients' receptiveness to alcohol or other lifestyle interventions [28], [29].

It has been suggested that ED presents a ‘teachable moment’ when patients may be more amenable to an intervention making a connection between alcohol consumption and the presenting problem, increasing motivation to reduce drinking [23]. Alternatively it is possible that patients make this connection by virtue of the distress of their presenting condition and having to attend ED, without it being pointed out by clinical staff, which might obviate the need for, and limit the potential impact of SBI [28], [29].

Nevertheless there is growing enthusiasm for implementation of SBI in ED in the UK and elsewhere [9], [30], [31]. A recent national survey of EDs in England conducted in 2011 reported that nearly half of EDs routinely ask patients about alcohol, 96% offer help or advice about alcohol, and 72% have access to an alcohol health worker or specialist nurse: significant increases on a 2006 survey [30]. Our results and the systematic review do not support widespread implementation of alcohol SBI in ED beyond the provision of screening followed by simple clinical feedback and alcohol information, which is likely to be easier and less expensive to implement than more complex interventions.

## Supporting Information

Protocol S1 **Trial protocol.**
(DOC)

Checklist S1 **CONSORT checklist.**
(DOCX)

## References

[1] Rehm J, Mathers C, Popova S, Thavorncharoensap M, Teerawattananon Y, et al (2009) Global burden of disease and injury and economic cost attributable to alcohol use and alcohol-use disorders. Lancet 373: 2223–2233. 19560604 10.1016/S0140-6736(09)60746-7

[2] Waller S, Thom B, Harris S, Kelly M (1998) Perceptions of alcohol related attendances in accident and emergency departments in England: a national survey. Alcohol Alcohol 33(4): 354–361. 9719393 10.1093/oxfordjournals.alcalc.a008404

[3] Drummond C, Phillips T, Coulton S, Barnaby B, Keating S, et al (2005) National prevalence survey of alcohol-related attendances at accident and emergency departments in England. Alcohol Clin Exp Res 29(5): 36A (suppl).

[4] French MT, Gumus G, Turner HL (2008) The role of alcohol use in emergency department episodes. Subst Use Misuse 43: 2074–2088. 18825591 10.1080/10826080802344849

[5] Cunningham RM, Bernstein SL, Walton M, Broderick K, Vaca FE, et al (2009) Alcohol, tobacco, and other drugs: future directions for screening and intervention in the emergency department. Acad Emerg Med 16: 1078–1088. 20053226 10.1111/j.1553-2712.2009.00552.x

[6] Kaner EFS, Dickinson HO, Beyer F, Pienaar E, Schlesinger C, et al (2009) The effectiveness of brief alcohol intervention in primary care settings: a systematic review. Drug Alc Rev 28: 301–323. 10.1111/j.1465-3362.2009.00071.x 19489992

[7] Bazargan-Hejazi S, Bing E, Bazargan M, Der-Martirosian C, Hardin E, et al (2005) Evaluation of a brief intervention in an inner-city emergency department. Ann Emerg Med 46: 67–76. 15988430 10.1016/j.annemergmed.2004.10.014

[8] Crawford MJ, Patton R, Touquet R, Drummond C, Byford S, et al (2004) Screening and referral for brief intervention of alcohol misusing patient in and emergency department: a pragmatic randomized controlled trial. Lancet 364: 1334–1339. 15474136 10.1016/S0140-6736(04)17190-0

[9] Dent AW, Weiland TJ, Phillips GA, Lee NK (2008) Opportunistic screening and clinician-delivered brief intervention for high-risk alcohol use among emergency department attendees: a randomized controlled trial. Emerg Med Austral 20: 121–8. 10.1111/j.1742-6723.2008.01067.x 18377401

[10] D'Onofrio G, Pantalon MV, Degutis LC, Fiellin DA, Busch SH, et al (2008) Brief intervention for hazardous and harmful drinkers in the emergency department. Ann Emerg Med 51: 742–750. 18436340 10.1016/j.annemergmed.2007.11.028 PMC2819119

[11] Cherpitel CJ, Korcha RA, Moskalewicz J, Swiatkiewicz G, Ye Y, et al (2010) Screening, brief intervention, and referral to treatment (SBIRT): 12 month outcomes of a randomised controlled trial in a Polish emergency department. Alc Clin Exp Res 34: 1922–1928. 10.1111/j.1530-0277.2010.01281.x PMC2965307 20659072

[12] D'Onofrio G, Fiellin DA, Pantalon MV, Charwaski MC, Owens PH, et al (2012) A brief intervention reduces hazardous and harmful drinking in emergency department patients. Ann Emerg Med 60: 181–192. 22459448 10.1016/j.annemergmed.2012.02.006 PMC3811141

[13] D'Onofrio G, Degutis LC (2002) Preventive care in the emergency department: screening and brief intervention for alcohol problems in the emergency department: a systematic review. Acad Emerg Med 9: 627–638. 12045080 10.1111/j.1553-2712.2002.tb02304.x

[14] Peters J, Brooker C, McCabe C, Short N (1998) Problems encountered with opportunistic screening for alcohol-related problems in patients attending an accident and emergency department. *Addiction* 93: 589–594. 9684397 10.1046/j.1360-0443.1998.93458914.x

[15] Drummond DC (1997) Alcohol interventions: do the best things come in small packages? Addiction 92: 375–379. 9177059

[16] Prime Minister's Strategy Unit (2004) Alcohol Harm Reduction Strategy. London, Cabinet Office.

[17] Kaner E, Bland M, Cassidy P, Coulton S, Dale V, et al (2013) Pragmatic cluster randomized controlled trial of the effectiveness and cost-effectiveness of screening and brief alcohol intervention in primary care in England. Br Med J 346: e8501. 23303891 10.1136/bmj.e8501 PMC3541471

[18] Newbury-Birch D, Bland M, Cassidy P, Coulton S, Deluca P, et al (2009) Screening and brief interventions for hazardous and harmful alcohol use in probation services: a cluster randomized controlled trial protocol. BMC Public Health 18: 418. 10.1186/1471-2458-9-418 PMC2784463 19922618

[19] Coulton S, Perryman K, Bland M, Cassidy P, Crawford M, et al (2009) Screening and brief intervention for hazardous alcohol use in accident and emergency departments: a randomized controlled trial protocol. BMC Health Serv Res 9: 114. 19575791 10.1186/1472-6963-9-114 PMC2712466

[20] Rollnick S, Mason P, Butler C (1999) Health Behaviour Change: A guide for practitioners. Edinburgh, Churchill Livingstone.

[21] Moyer A, Finney JW, Swearingen CE, Vergun P (2002) Brief interventions for alcohol problems: a meta-analytic review of controlled investigations in treatment-seeking and non-treatment-seeking populations. Addiction 97: 279–292. 11964101 10.1046/j.1360-0443.2002.00018.x

[22] Wittes J (2002) Sample size calculations for randomized controlled trials. Epidemiol Rev 24 (1): 39–53. 10.1093/epirev/24.1.39 12119854

[23] Williams S, Brown A, Patton R, Crawford MJ, Touquet R (2005) The half-life of the ‘teachable moment’ for alcohol misusing patients in the emergency department. Drug Alc Dep 77: 205–208. 10.1016/j.drugalcdep.2004.07.011 15664722

[24] Blow FC, Ilgen MA, Walton MA, Czyz EK, McCammon R, et al (2009) Severity of baseline alcohol use as a moderator of brief interventions in the emergency department. Alcohol Alcohol 44: 486–490. 19692345 10.1093/alcalc/agp031 PMC2765353

[25] Walton MA, Goldstein AL, Chermack ST, McCammon RJ, Cunningham RM, et al (2008) Brief alcohol intervention in the emergency department: moderators of effectiveness. J Stud Alcohol Drugs 69: 550–560. 18612571 10.15288/jsad.2008.69.550 PMC3646582

[26] Bernstein JA, Bernstein E, Heeren TC (2010) Mechanisms of change in control group drinking in clinical trials of brief alcohol intervention: implications for bias toward the null. Drug Alc Rev 29: 498–507. 10.1111/j.1465-3362.2010.00174.x 20887573

[27] Daeppen J-B, Gaume J, Bady P, Yersin B, Clames J-M, et al (2007) Brief alcohol intervention and alcohol assessment do not influence alcohol use in injured patients treated in the emergency department: a randomized controlled clinical trial. Addiction 102: 1224–1233. 17565563 10.1111/j.1360-0443.2007.01869.x

[28] Field CA, Baird J, Saitz R, Caetano R, Monti PM (2010) The mixed evidence for brief intervention in emergency departments, trauma centres, and inpatient hospital settings: what should we do? Alc Clin Exp Res 34: 2004–2010. 10.1111/j.1530-0277.2010.01297.x PMC2988943 20860610

[29] Trinks A, Festin K, Bendtsen P, Nilsen P (2013) What makes emergency department patients reduce their alcohol consumption? a computer based intervention study in Sweden. Int Emerg Nurs 21(1): 3–9. 23273798 10.1016/j.ienj.2011.11.004

[30] Patton R, O'Hara P (2012) Alcohol: signs of improvement. The 2^nd^ national emergency department survey of alcohol identification and intervention activity. Emerg Med J doi:10.1136/emermed-2012-201527 10.1136/emermed-2012-201527 22878039

[31] Cunningham RM, Harrison SR, McKay MP, Mello MJ, Sochor M, et al (2010) National survey of emergency department alcohol screening and intervention practices. Ann Emerg Med 55: 556–562. 20363530 10.1016/j.annemergmed.2010.03.004

