# Supplementary material for: The Effectiveness of Alcohol Screening and Brief Intervention in Emergency Departments: A Multicentre Pragmatic Cluster Randomized Controlled Trial
Source: PLoS One. 2014 Jun 25;9(6):e99463. doi: 10.1371/journal.pone.0099463 (PMC4070907; doi:10.1371/journal.pone.0099463)
Supplement: Protocol S1 — Trial protocol. (DOC) [file pone.0099463.s001.doc]

**REC ref.:** 07/MRE02/6

**Short Title:** SIPS - AED

**Document name:** Research protocol

**Version:** **5**

**Date: 04/04/09**

# Institute of Psychiatry, King’s College London – Newcastle University – St George’s, University of London - University of York – Imperial College, University of London – Alcohol Concern

**A Randomised Controlled Trial of Different Methods of Alcohol Screening and Brief Interventions in Routine Accident and Emergency Department Care**

**Research Protocol**

Synopsis

This pragmatic trial with cluster randomization of AEDs is designed to evaluate the effectiveness and cost-effectiveness of screening methods (modified PAT vs FAST vs modified SASQ) and interventions (Leaflet vs Brief advice vs Brief counselling) in routine AED care. Due to the design implications of a multi-level factorial design in AEDs the proposed trial involves a universal screening approach in which relevant data for a sub-sample of linked presentation population is collected. Therefore, the trial design encompasses a 3x3 factorial trial, screening method (modified PAT vs FAST vs modified SASQ) and intervention (Leaflet vs Brief advice vs Brief lifestyle counselling). The main advantages of utilising a factorial approach are twofold. First each of the three elements (screening tool and intervention) can be analysed independently with sufficient power to make meaningful interpretation of relative effectiveness. Second, the method enables meaningful interpretations of the relative effectiveness of any combination of screening method and intervention. Because the universal screening population encompasses a sub-group of linked presentation targeted population we can make meaningful comparisons of the relative effectiveness and interactions associated with screening approach in AEDs. Nine AEDs across the North East, London and the South East Regions of England will be recruited. AEDs will be randomly allocated to one of three intervention conditions: the patient information leaflet control condition (n=3), the structured brief advice condition (n=3); and lifestyle counselling by an Alcohol Health Worker (n=3). To test the relative effectiveness of different screening methods all AEDs will be randomised to either a modified single item screen (Single Alcohol Screening Question; M-SASQ) or a modified Paddington Alcohol Test (SIPS-PAT) or FAST Alcohol Screening Test (FAST) screening method. AEDs in the modified PAT condition will use targeted screening as per the standard PAT protocol. AEDs in the FAST and modified SASQ conditions will use universal screening for case identification and collect information about whether a patient would be part of a linked presentation group and then use statistical simulation to analyse any interactions. Screening randomisation will be stratified by median AED admission rates (2 levels) and intervention condition (3 levels). Each AED will recruit a minimum of 131 patients who are hazardous or harmful drinkers into the trial and a short baseline assessment will be carried out. Outcome will be assessed at 6, and 12 months after intervention. Outcomes will include system implementation outcomes and patient outcomes. We will also examine the practitioner and organisational factors associated with successful implementation.

Study aim

To evaluate the implementation, effectiveness and cost effectiveness of BI delivered by an alcohol health worker aimed at reducing excessive drinking in routine AED care compared to brief advice and a patient information leaflet (PIL) in hazardous and harmful drinkers identified by universal or targeted screening.

Study objectives

- To conduct a pragmatic multicentre cluster randomised controlled trial of screening and brief interventions for hazardous and harmful drinkers in AEDs in three English regions.
- To compare the effectiveness and cost effectiveness of brief advice conducted by AED staff (including dedicated AHWs) (Tier 1) with referral to an alcohol health worker (Tier 2) of patients with hazardous and harmful alcohol consumption identified by targeted opportunistic or universal screening.
- To assess the relative impact of the three implementation strategies on alcohol screening and brief intervention activity in AEDs.
- To identify the attitudinal, practical, skill, resource, and reinforcing factors that predict successful implementation of screening and brief intervention in AEDs.
- To identify the optimal method of alcohol screening in AED.
- To assess the relative impact of the three implementation strategies on uptake of alcohol services, including an alcohol helpline.

Hypotheses

- Brief Lifestyle Counselling intervention by an alcohol health worker for hazardous and harmful drinkers identified by targeted screening is more effective and cost effective than Brief Advice conducted by AED staff (including dedicated AHWs) in the typical AED setting.
- Brief Lifestyle Counselling intervention by and alcohol health worker and Brief Advice by AED staff (including dedicated AHWs) are more effective and cost effective than an information leaflet alone.
- Access to referral to an alcohol health worker results in greater screening and intervention activity than training of AED staff in screening and brief intervention alone.
- Attitudinal, practical, skill, resource, and reinforcing factors predict screening and intervention activity.
- Briefer screening methods result in greater implementation of screening activity than more complex methods.

Setting

Nine AEDs in three English regions (North East, London, South East) will be recruited to take part in the study. Recruitment will take place with the assistance of the British Association of Accident and Emergency Medicine (BAEM, of which Prof Touquet is a Professor of Accident and Emergency Medicine and adviser to BAEM on the management of alcohol misuse). All AEDs in the three regions that do not have current routine alcohol screening and brief intervention facilities will be eligible to participate. AEDs will be selected to provide a range of both urban and rural catchment areas.

Design

AEDs will be randomly allocated to one of three implementation conditions (leaflet, brief advice or brief lifestyle counseling intervention) in a cluster randomized design such that there will be 3 AEDs in each condition. AEDs will be randomized to one of three screening conditions (described below) such that there will be 3 AEDs in each condition (see Table **1**). Patients enrolled in the study will have a baseline research assessment carried out by structured interview, and followed in parallel groups over 6, and 12 months. Outcome assessment will be by face to face or telephone interview conducted by a researcher.

The AED study design differs from the PHC study in not having targeted versus universal screening as a separate factor in the design. There are two main reasons for this. First, we have looked closely at the potential of a factorial design including universal versus targeted screening. However, the number of AEDs that would be required to have a separate screening factors (minimum n=18) would exceed the likely number of AEDs agreeing to participate in the there regions (based on previous experience from our national AED survey (Drummond et al., 2003)). Second the PAT has been designed as a targeted screening method in the context of routine AED assessment. The FAST and SASQ on the other hand have been designed as universal screening tools. Therefore we propose that universal versus targeted screening be studied in two ways within this trial. First we will randomize AEDs to targeted screening with the modified PAT and universal screening with FAST or the modified SASQ, which is appropriate to the design of the instruments. Second we will record the patients’ presenting clinical conditions and demographic factors in the universal screening arms of the study to identify which factors are most strongly associated with alcohol positive cases. Together these findings will allow the comparison of targeted and universal screening and lead to the development of clinical guidelines for AEDs on the most efficient form of targeted screening approach.

Table **1**. Overall AED study design.

| Intervention condition **>** | Leaflet by AED staff | Brief advice by AED staff | Brief Lifestyle  Counselling intervention | Totals |
| --- | --- | --- | --- | --- |
| Screening condition V |
| FAST Alcohol Screening Test | AED n=1;  Patients n=131 | AED n=1;  Patients n=131 | AED n=1;  Patients n=131 | AED n=3;  Patients n=393 |
| Modified Single Alcohol Screening Question | AED n=1;  Patients n=131 | AED n=1;  Patients n=131 | AED n=1;  Patients n=131 | AED n=3;  Patients n=393 |
| SIPS Paddington Alcohol Test | AED n=1;  Patients n=131 | AED n=1;  Patients n=131 | AED n=1;  Patients n=131 | AED n=3;  Patients n=393 |
| Total per intervention | AED n=3  Patients n=393 | AED n=3  Patients n=393 | AED n=3  Patients n=393 | AED n=9  Patients n=1179 |

Screening conditions

In both intervention conditions the research team and specialist clinicians will train and support all participating AEDs in implementing screening systems tailored to the operational model of the AED. Where possible computerized screening tools will be developed to be embedded in AED computerized records systems.

In order to test the relative effectiveness of different screening methods in identifying hazardous and harmful drinkers we intend to conduct a cluster randomized comparison of the modified Paddington Alcohol Test (PAT) (adapted from Patton et al, 2004), the FAST Alcohol Screening Test (FAST; Hodgson et al., 2002) and the modified single item screening test, Single Alcohol Screening Question (SASQ) (adapted from Canagasaby & Vinson, 2005): “How often do you have X or more standard drinks on one occasion?”, where X = 6 for women and 8 for men, with monthly, or weekly, or daily or almost daily considered a positive screen (1 drink = unit of alcohol). The original SASQ has been adapted to the UK’s standard drinks and validated during our pilot research. The new question has a higher sensitivity and specificity then the original SASQ when compare to the gold standard Alcohol Use Disorders Identification Test (AUDIT).

FAST has undergone validity testing in AED settings as a universal screening tool and has been found to be of high sensitivity and specificity (Hodgson et al., 2002) and performs well in comparison to the currently recognised ‘gold standard’ the Alcohol Use Disorders Identification Test or AUDIT.

PAT has been simplified to fit more with the pragmatic nature of this trial by focussing on two questions that have emerged in previous studies (Patton et al., 2004) with high sensitivity and specificity whilst also retaining it’s targeted screening feature.

However, it is unclear which approach is most effective in identifying cases in the typical AED setting. The modified PAT (SIPS-PAT) is a targeted screening tool which is applied in cases with presenting conditions commonly associated with alcohol misuse. The original PAT has been used at St Mary’s Hospital embedded in the standard clinical assessment process. SASQ on the other hand has been studied in PHC and, based on our pilot research, is more likely to be adopted by busy AED staff. The FAST has been used mainly in a research screening context to establish prevalence rather than as a clinical tool. Also this single question in the modified SASQ will identify individuals whose level of drinking is more likely to lead to AED attendance, and is the same first item of the FAST questionnaire, which was found to identify 50% of hazardous and harmful drinkers (Hodgson et al., 2002).

Brief intervention implementation conditions

1. *Control condition:* AED staff (including dedicated AHWs) in the control condition will be trained to apply the appropriate screening method and record the screening outcome, will feedback the result of screening to patients and offer them a patient information leaflet (PIL. The PIL from the Drink-Less Programme and has been extensively pre-tested with clinicians and patients in PHC and will be used in this trial. The PIL will also contain a number for Drinkline where the patient can access further information). The PIL to be used in this trial will be ‘Alcohol and Sensible Drinking’ as in the PHC study.

2. *Brief Advice condition:* AED staff (including dedicated AHWs) will be trained to carry out screening and deliver up to 5 minutes of simple structured brief advice for hazardous and harmful drinkers presenting to the AED, using the Drink-Less brief intervention materials (level 1) as in the PHC study. Patients in this condition will also receive a Patient Information Leaflet (PIL) as above, including a number for Drinkline.

3. *Brief Lifestyle condition:* This is based on the St Mary’s Hospital model. AED staff (including dedicated AHWs) will be trained to carry out universal alcohol screening and to refer hazardous and harmful drinkers identified by screening to an Alcohol Health Worker (AHW), by making an appointment usually the following day or as soon as possible after AED attendance. Before leaving the AED the patients in this condition will be given a PIL and Simple structured advice as above. The AHW will be experienced in carrying out alcohol assessment and brief interventions. The AHW will carry out a brief lifestyle counseling intervention lasting for 20 minutes using the modified Drink-Less brief intervention materials (level 2).

AED recruitment

Eligible AEDs in the participating regions will be contacted initially by phone and mail to canvass participation in the study. We will use contacts previously made in our national survey of AEDs (Drummond et al., 2003) and through the BAEM with Prof Touquet’s assistance. Thereafter site visits will be made by the research team to explain the trial protocol, secure clinician consent to participate in the study and to organize training.

Training and support

All participating AEDs will be provided with training and support appropriate to the allocated condition in the study. In the control condition staff will be trained by experienced SBI clinical trainers to carry out screening, feedback and provide a PIL. A member of the research team and a specialist clinician will provide ongoing support in implementing the research and clinical procedures.

In the Brief Advice condition training and support will be provided by a specialist clinician (Alcohol Health Worker) who will act as clinical coordinator for the study, and a member of the research team. Training will be carried out by experienced trainers for medical, nursing and administrative staff in seminars and individual training (see ‘incentives’ below). Trainers will be Alcohol Health workers recruited from local alcohol agencies. Each AED will have an allocated clinical coordinator who will champion screening and brief intervention and provide ongoing support and supervision for AED staff, including an AED staff support group. The research coordinator will provide support on implementation of the research procedures.

In the Brief Lifestyle Counselling condition the training and support for AED staff will be the same as in the simple structured advice condition except that staff will be trained to screen and refer to the AHW. The equivalent of approximately 0.5 WTE AHW salary per AED has been allocated. In some areas it may be more practical to have one AHW split between two AEDs. In other areas different configurations can apply. The AHW will be responsible for training and supporting the AED staff in implementing screening and referral over the course of the project. The AED staff will also be supported in implementation of the screening and research procedures by a member of the research team.

Inclusion criteria

AEDs: Eligible AEDs will be those not already providing routine screening and SBI.

Patients: Any AED patients scoring positive on the modified PAT, FAST or modified SASQ aged 18 or over, and who are alert and orientated, resident within 20 miles and able to speak, read and write English sufficiently well to complete study questionnaires.

Exclusion criteria

Those who are currently seeking help for alcohol problems and those who are involved in any alcohol research study will be excluded. Any patients within the AED setting who are severely injured, suffering with a serious mental health problem and/or who are grossly intoxicated will also be excluded from the study. Finally patients with no fixed abode will be excluded from the study.

Consent

Consent to participate will be obtained in a 2 stage process. AED staff (including dedicated AHWs) will initially establish verbal consent to be check eligibility to take part, collect some basic demographic information and to be screened. Those who then are positive on FAST or modified SASQ or SIPS-PAT as applicable, will have the study explained to them verbally by AED staff (including dedicated AHWs) and in writing (using the patient information sheet). Written informed consent will be obtained by AED staff (including dedicated AHWs). This will include permission to give the patient's data and contact details to the research staff, and provide the research team with access to the patients AED records, and to participate in follow up after 6, and 12 months. The research team will then contact the patient within two weeks to thank him/her to take part in the study.

Measures

Staff attitudinal and organisational measures:

Medical and nursing staff in the participating AEDs will be surveyed before and after training in each condition of the study using an attitudinal measure initially developed by Green et al. (1988) and further developed in the study by Babor et al. (2005). The factors found to be relevant to implementation of screening and brief intervention are divided into *predisposing*, *enabling* and *reinforcing* factors. Predisposing factors are mainly attitudinal that relate to clinicians’ willingness to implement screening and brief intervention. Enabling factors are the skills and resources needed to implement screening and brief intervention, and reinforcing factors are visible results, feedback from peers and patients and other factors that encourage continuation of screening and brief intervention. Babor et al (2005) collected data on these factors in two ways: (1) surveys of providers and specialists completed prior to training, after training and at the end of project operations (five items, Babor et al., 2004) and (2) independent ratings by two research staff based on information recorded from regular technical assistance contacts and site visits throughout the implementation process (17 items). Inter-rater reliability in Babor et al’s study was high for all factors (median *r* = 0.70).

In Babor’s study predisposing factors were (1) Peer approval for alcohol screening. (2) Organizational approval for alcohol screening. (3) Frequency clinicians asked about alcohol consumption. (4) Frequency clinicians educated patients about health risks. (5) Frequency clinicians advised patients with problem drinking to cut down or stop drinking. (6) Stable patient membership was based on research staff ratings of each site as to whether the patient membership was stable or changing. (7) Organisational instability was based on ratings of each site in terms of fiscal and management stability. Enabling factors considered as enabling implementation were derived from the ratings conducted by the two research staff. These factors were (8) number of clinicians trained at each clinic, (9) practitioner lack of time, (10) nursing staff lack of time, (11) receptionist staff lack of time, (12) practitioner turnover, (13) nursing staff turnover, (14) receptionist staff turnover, (15) competing organizational priorities, (16) influential site coordinator, (17) involvement of clinic staff in planning, (18) facilitation by computer technology, (19) amount of technical assistance and (20) successful procedural changes to implement screening and interventions. The two factors classified as reinforcing implementation of were also derived from the research staff ratings. Organisational support (21) and financial incentives (22). Last, an average organizational score was created based on the sum of the 17 item the two researchers rated. This score indicated the total extent of favourable predisposing, enabling and reinforcing factors observed at a given clinic.

In this study we intend to survey AED staff before and after training and compare these factors between different implementation models. We also intend to carry out independent ratings of organisational factors as in the model described by Babor et al (2005) during the course of the implementation phase of the study.

A list of all professional staff that can deliver alcohol screening and brief intervention in each study site will be compiled. A self-administered questionnaire will be distributed to participating staff on three occasions: pre training, post training and post study (see enclosed AED Staff T3 questionnaire). All three questionnaires will contain called the shortened version of the Alcohol and Alcohol Problem Perception Questionnaire (SAAPPQ: Gorman and Cartwright 1991) which assesses staff attitudes towards working with problem drinkers. The SAAPPQ has five subscales - Role adequacy, role legitimacy, self-esteem, motivation, and work satisfaction. Role adequacy and role legitimacy are concerned with role security i.e., how individual’s perceive the adequacy of their skills and knowledge in relation to problem drinkers and how appropriate it is for them to work with such clients. The other subscales, self esteem, motivation and work satisfaction are concerned with worker’s therapeutic commitment, i.e., the extent to which they seek to engage drinkers in treatment and the extent that they find the work rewarding on both a professional or personal level (Gorman and Cartwright 1991)

In addition to the SAAPPQ the post training and post study questionnaire will contain a number of semi-structured questions developed to elicit information on staff attitudes towards alcohol screening and brief intervention; previous experience of delivering alcohol screening and brief intervention; readiness to undertake these activities; the training needed to conduct screening and brief intervention; the suitability of each site to provide SBI; and potential barriers to effective implementation.

**A further qualitative study aimed at exploring more in-depth the experiences of practitioners involved in the delivery of screening and brief intervention in routine practice will also be carried out with selected staff (see enclosed AED Protocol Qualitative Study V1).**

System measures:

The research team will identify the total number of patients over 18 attending each AED during the recruitment period, the total number of patients screened, the number screening positive, and the number receiving an alcohol intervention (either a leaflet or brief advice by AED staff (including dedicated AHWs) or brief lifestyle counselling intervention by the AHW) according to the method described by Babor et al. (2005). This will allow calculation of the overall screening rate, the screen conversion rate (proportion of positive screens), and the intervention rate in the different settings. We will also compare these measures between AEDs assigned to the SIPS-PAT versus the modified SASQ versus FAST screening methods.

Reattendence to AEDs over the 6, and 12 month follow up period of patients participating in the study will be assessed using computerised admission records and compared with admissions by the participating group of patients in the 6 month before entry into the study. The sustainability of the screening and intervention approaches will be assessed by examining the extent to which screening and intervention activity continues after the end of the formal study recruitment period.

Patient measures:

*Baseline*

Before receiving the initial PIL and/or brief advice intervention and/or brief lifestyle counselling intervention, participants will be invited by AED staff (including dedicated AHWs) to provide the contact details and complete the Extended AUDIT, EQ5D, Short-SUQ and modified Readiness to change Ruler. Participants in the extended intervention will complete the AUDIT at the same stage as those in other groups.

The Alcohol Use Disorders Identification Test (AUDIT: Saunders et al. 1993) is normally used as a screening test for alcohol use disorders. However in this context the AUDIT will be used as a means of establishing the severity of alcohol use disorders at baseline, in a way that is least intrusive to naturalistic aim of the trial in the AED setting, and as a means of measuring the adequacy of matching between the intervention groups at baseline. The AUDIT contains 10 items to measure alcohol consumption, alcohol problems and dependence over, in this case, the previous 6 months, and the sum of the item scores provides a measure of severity, which has been used in several previous studies, allowing comparability with other AED samples (Drummond et al., 2005 [ANARP]). We are concerned that the use of more elaborate baseline alcohol consumption measures would interfere with the naturalistic aims of the study and possibly would contribute a form of intervention in themselves, so introducing bias into the evaluation of the interventions by reducing the difference between trial interventions. In addition, participants will complete the Euroqol (EQ5D) as a brief 5 item measure of quality of life. Use of health, social criminal justice services and wider societal costs will be measured via a shortened version of the Service Use Questionnaire (SUQ) which allows estimation of health care and wider social costs for health economic analysis in the six months prior to intervention. A modified version of the Readiness Ruler (LaBrie et al., 2005) will assess participants’ motivational state to change their drinking behaviour.

*Follow-up*

At 6 and 12 months after intervention, all patients will be contacted either by telephone, or by post, or by email, based on their preference expressed at baseline, by research staff who will be blind to their intervention condition. Patients might also be offered a face-to-face follow-up if preferred. Researchers will administer the shorter Alcohol Use Disorders Identification Test (Extended AUDIT). Alcohol-related problems will be assessed via the brief Alcohol Problems Questionnaire (APQ; Drummond, 1990; Williams & Drummond, 1994). Use of health, social criminal justice services and wider societal costs will be measured with the Service Use Questionnaire (SUQ) which has been used in Primary health care settings (Drummond et al., 2003). This questionnaire allows estimation of health care and wider social costs for health economic analysis in the six months prior to intervention. The Euroqol (EQ5D) will be included as a brief 5 item measure of quality of life, and the modified Readiness to Change ruler will assess motivation to change drinking behaviour.

At follow-up, each patient will also be asked if, and how often, they made use of the Drink-Line telephone number. At 12-month follow up an additional 6-item patient satisfaction questionnaire will also be asked (PSQ) to understand how people felt about the help or advice they received as part of this study.

Economic evaluation

The economic component of the study comprises a cost-effectiveness and cost-utility analysis. The study aims to identify, quantify and value resources related to alcohol SBI by clinicians in AEDs and the subsequent use of health, social care, and criminal justice services by patients following each type of intervention.

Resources utilised in the identification and brief intervention delivery or control condition will be recorded by AED staff (including dedicated AHWs) involved on an ongoing basis. This will allow the calculation of costs related to implementation of different models of screening and brief intervention. Local costs will be used to calculate the costs of the interventions, which will include staff costs, premises costs and costs of leaflets and other consumables. In addition, specific training costs for staff will be calculated, in terms of staff time, premises costs and the cost of training materials.

Patients’ use of health, social care and criminal justice services will be identified retrospectively using a short form of the Service Use Questionnaire and applying a common set of national unit cost estimates. Patient costs in the 6 month period before SBI can then be compared to cost in the 6 month period after SBI to explore any changes in costs imposed by patients in each group.

The economic analysis will calculate the incremental cost-effectiveness of the control condition with the AHW condition under study, using measures of clinical outcome and quality of life (EQ-5D; The EuroQoL Group, 1990) responses at baseline and at 6 and 12 month follow ups. The use of EQ-5D enables the estimation of Quality Adjusted Life Years. Data will be bootstrapped to account for the expected skewness evident in economic cost data (National Institute for Clinical Excellence, 2004). The analysis will include the construction of cost-effectiveness acceptability curves to illustrate the probability that the brief intervention is more cost-effective than usual care, based on different monetary values being attached to QALYs. The use of QALYs follows the recommendations of NICE (Briggs & Gray, 1999) and enables the value for money afforded by treatment to be compared to a range of other health care interventions. Furthermore, combination of the economic cost data and outcome data with patient data collected in the trial will enable a secondary analysis of various patient characteristics that may influence the cost-effectiveness of the intervention.

Incentives

We intend to use funding from the project budget (£250,000 salary costs for 4 x FTE AHW) to provide incentives for AEDs to participate. We have made informal enquiries with AED consultants and staff in the regions we intend to recruit from and have reached the following conclusions. Effective incentives for AED participation will be very different from PHC. AEDs do not have an equivalent fee for service or capitation structure. Therefore we have budgeted for 4 WTE AHW staff across the 9 participating AEDs. The AED role will be different for AEDs in different implementation conditions. However, whichever implementation condition the AED is in, the AHW will provide the appropriate training, support, and (in the case of the AHW condition) clinical intervention. The equivalent of approximately 0.5 AHW salary per AED has been allocated. In some areas it may be more practical to have one WTE AHW split between two AEDs. In other areas different configurations can apply. This is appropriate to the typical existing role of AHWs/Alcohol Liaison Nurses. More time will be allocated to providing interventions to AEDs in the AHW condition. The AHW will work closely with the members of research team allocated to the respective AEDs in implementing to project at their allocated sites.

Participants will receive a £10 voucher, together with a Thank You letter, in the post shortly after completing the baseline research interview and another £10 voucher for completing each of the research follow-up interviews. No payments will be made to participants for undertaking screening or interventions.

Sample size calculation

The sample size calculation is designed to account primarily for intervention level outcomes. Powering the study in this way will also account statistically for appropriate outcomes for screening approach and screening method. The primary outcome for this study is the proportion of patients who consume alcohol within recommended levels at 6 month follow up. Recent meta-analysis (Moyer et al 2002) suggest that the difference between brief intervention and control is of the order 13%, 5% reduction in the control group and 18% in the brief intervention group. In order to detect a difference of this magnitude at the 5% significance level with 80% power, for a 2-sided test, requires 109 patients in each of the 3 groups, a total of 327. Our experience with other multi-centre randomized controlled trials of interventions for alcohol use disorders suggests that with assiduous follow-up the potential loss to follow-up across groups is of the order 25%. Taking this loss into accounts inflates the sample required to 131 in each group, a total of 393 patients per intervention.

The proposed study involves a cluster design and requires a statistical adjustment to account for any potential cluster effect. The literature, and our previous experience of trials in primary care, suggest an intra-class correlation coefficient of 0.04 is appropriate. Assuming a cluster size of the order 131 patients this inflates the sample size calculation by a factor of 2.7 requiring patients 393 in each group, a total of 1179, with an expectation that at least 882 will be followed up at 6 months.

We propose to recruit 9 AED’s. AED populations will be screened using 1 of 3 screening method groups (3 AED’s in each group) and will receive 1 of 3 intervention (3 AED’s in each group).

Planned analysis

As the study is pragmatic in design, the planned analysis will be by intention to treat. The primary outcome is dichotomous in nature, drinking within or above recommended levels, and will be analysed logistic regression adjusting for all known prognostic factors, data will be presented as odds ratios and their corresponding confidence intervals. Secondary analyses will be undertaken using the appropriate method for the outcomes, controlling where appropriate for intake values and other known prognostic variables using analysis of covariance. Simulations will be undertaken to encompass both screening approach groups and intention to treat analysis will be undertaken on both groups.

Due to the nested factorial nature of the study, we will use multi-level modelling to explore potential interactions between each of the levels nested within the trial.

Practice and patient factors will be utilised as part of regression model to explore possible prognostic factors that impact on outcome. Interaction analysis will explore any possible interactions between practice and patient characteristics and outcome. The efficacy of the interventions will be explored with a secondary analysis utilising a per protocol approach. A sub-group of the trial population, those who engaged in the allocated treatment will be utilised for this analysis.

Ethical and Research Governance Approval

We will seek Multi-centre ethical approval for the trial plus local agreement from all relevant LRECS. In addition, research governance approval will be sought from all relevant acute NHS trusts.

Project Timescales and Funding

The trial duration will be 2 years beginning at the point that ethics and governance approval has been secured.

**REFERENCES**

Aalto, M., Pekuri, P. & Seppa, K. (2003). Obstacles to carrying out brief intervention for heavy drinkers in primary health care: a focus group study. *Drug & Alcohol Review*, 22, 169-173.

Academy of Medical Sciences (2004). *Calling Time: the Nation’s Drinking as a Major Health Issue.* London: Author.

Alaszewski, A. & Harrison, L. (1992). Alcohol and social work: a literature review. *British Journal of Social Work,* 22**,** 331-343.

Alcohol Concern (2002). *Report on the Mapping of Alcohol Services.* London: Alcohol Concern

Anderson, P. & Scott, E. (1992). The effect of general practitioners' advice to heavy drinking men. *British Journal of Addiction,* 87, 891-900.

Anderson, P., Laurant, M., Kaner, E., Wensing, M., Grol, R. (2004a) Engaging general practitioners in the management of hazardous and harmful alcohol consumption: results of a meta-analysis. *Journal of Studies on Alcohol*, 65: 191-199.

Anderson, P., Kaner, E., Wutzke, S., Wensing, M., Grol, R., Heather, N., et al. (2003). Attitudes and management of alcohol problems in general practice: descriptive analysis based on findings of a World Health Organization international collaborative study. *Alcohol & Alcoholism,* 38, 597-601.

Anderson, P., Kaner, E., Wutzke, S., Funk, M., Heather, N., Wensing, M., et al. (2004b). Attitudes and managing alcohol problems in general practice: an interaction analysis based on findings from a WHO Collaborative Study. *Alcohol & Alcoholism,* 39, 351-359.

Babor, T.F., Higgins-Biddle, J., Dauser, D., Higgins, P. & Burleson, J.A. (in press). Alcohol screening and brief intervention in primary care settings: implementation models and predictors. *Journal of Studies on Alcohol.*

Babor, T. F. & Higgins-Biddle, J. (2000). Alcohol screening and brief intervention: dissemination strategies for medical practice and public health. *Addiction*, 95, 677-686.

Babor, T.F., Higgins-Biddle, J.C., Higgins, P.S., Gassman, R.A. and Gould, B.E. (2004) Training medical providers to conduct alcohol screening and brief interventions. *Substance Abuse* 25: 17-26.

Babor, T.F. and Grant, M. (1992) *Project on Identification and Management of Alcohol Related Problems. Report on Phase II: A Randomized Clinical Trial of Brief Interventions in Primary Health Care*, Geneva, Switzerland: World Health Organization.

Ballesteros, J., Duffy, J. C., Querejeta, I., Arino, J. & Gonzalez-Pinto, A. (2004). Efficacy of brief interventions for hazardous drinkers in primary care: systematic review and meta-analysis. *Alcoholism: Clinical & Experimental Research,* 28, 608-618.

Barnaby, B., Drummond, D.C., McCloud, A., Omu, N. & Burns, T. (2003) Substance misuse in psychiatric inpatients: comparison of a screening questionnaire survey with case notes. *British Medical Journal*, 327, 783-784.

Barrett, B., Byford, S., Crawford, M.J., Patton, R., Drummond, C., Henry, J.A. & Touquet, R. (2005) Cost-effectiveness of screening and referral to an alcohol health worker in alcohol misusing patients attending an accident and emergency department: a decision-making approach. *Drug and Alcohol Dependence*, in press.

BCS (2002) British Crime Survey, Home Office.

Beich, A., Gannik, D. & Malterud, K. (2002). Screening and brief intervention for excessive alcohol use: qualitative interview study of the experiences of general practitioners. *British Medical Journal,* 325, 870-872.

Beich, A., Thorsen, T. & Rollnick, S. (2003). Screening in brief intervention trials targeting excessive drinkers in general practice: systematic review and meta-analysis. *British Medical Journal,* 327, 536-542.

Bertholet, N., Daeppen, J-B., Wietlisbach, V., Fleming, M. & Burnand, B. (in press). Brief alcohol intervention in primary care: systematic review and meta-analysis. *Archives of Internal Medicine.*

Best D., Noble A., Stark MM., Marshall EJ. (2002). The role of forensic medical examiners and their attitudes on delivering brief alcohol interventions in police custody. *Criminal Behaviour and Mental Health,* 12, 230-235

Bien, T. H., Miller, W. R. & Tonigan, J. S. (1993). Brief interventions for alcohol problems: a review. *Addiction,* 88, 315-335.

Briggs, A.H., Gray, AM. (1999) Handling uncertainty when performing economic evaluation of health care interventions. *Health Technology Assessment*; **3** (2).

British Medical Association/ The NHS Confederation (2004). *The New GMS Contract: Supplementary Documents*. London: BMA.

Budd T, Sharp C & Mayhew P (2005). *Offending in England and Wales: First results from the 2003 Crime and Justice Survey.* Home Office Research Study 275. London, Home Office Research Development and Statistics Directorate.

Campbell, M.K., Grimshaw, J.M., Steen, I.N. (2000) Changing Professional Practice in Europe Group. Sample size calculations for cluster randomised trials. *Journal of Health Service Research Policy*; 5: 12-6.

Canagasaby, A. & Vinson, D.C. (2005). Screening for hazardous or harmful drinking using one or two quantity–frequency questions. *Alcohol and Alcoholism*, 240(3):208-213.

Centre for Drug and Alcohol Studies. (1993) *The Drink-Less Programme*. Sydney: Department of Psychiatry, Sydney University, Australia.

Chick J., Lloyd G., & Crombie E. (1985) Counselling problem drinkers in medical wards: a controlled study. *British Medical Journal*, 290, p. 965–967.

Coulton, S., Drummond, C., James, D., Godfrey, C., Parrott, S. & Peters, T. (2005) Opportunistic screening for alcohol use disorders in primary care: a comparison of screening methods. *British Medical Journal,* Submitted.

Crawford, M.J., Patton, R., Touquet, R., Drummond, C., Byford, S., Barrett, B., Reece, B., Brown, A., Henry, J.A. (2004) Screening and referral for brief intervention of alcohol misusing patients in an Accident and Emergency Department: a pragmatic randomised controlled trial*. The Lancet* 364, 1334-39.

Cuijpers, P., Riper, H. & Lemmens, L. (2004). The effects on mortality of brief interventions for problem drinking: a meta-analysis. *Addiction,* 99, 839-845.

D’Onofrio, G., Nadel, E.S., Degutis, L.C., Sullivan, L.M., Casper, K. et al (2002). Improving emergency medicine residents’ approach to patients with alcohol problems: A controlled educational trial. *Annals Emergency Medicine.* 40, 50-62.

D'Onofrio, G. & Degutis, L.C. (2002). Preventive care in the emergency department; screening and brief intervention for alcohol problems in the emergency department: a systematic review. *Academic Emergency Medicine,* 9, 627-638.

Deehan, A., Templeton, L., Taylor, C., Drummond, D.C. & Strang, J. (1998a) How do general practitioners manage alcohol misusing patients? Results from a national survey of GPs in England and Wales. *Drug and Alcohol Review,* 17(3), 259-266.

Deehan, A., Templeton, L., Taylor, C., Drummond, D.C. & Strang, J. (1998b) Are practice nurses an unexplored resource in the identification and management of alcohol misuse: Results from a study of practice nurses in England and Wales in 1995. Journal of Advanced Nursing, 28(3), 291-298.

Deehan, A., Templeton, L., Taylor, C., Drummond, C. & Strang, J. (1998c). Low detection rates, negative attitudes and failiure to meet the 'Health of the Nation' alcohol targets: findings from a national survey of GPs in England and Wales. *Drug & Alcohol Review*, 17, 249-258.

Deehan, A, Marshall, E and Saville E (2002) Drunks and Disorder: processing

intoxicated arrestees in two city-centre custody suites. Home Office Police Research

Series Paper 150 (www.homeoffice.gov.uk/rds/prgpdfs/prs150.pdf)

Department of Health (1993) *Health of the Nation Key Area Handbook on Accidents*. HMSO, London.

Department of Health (2001) *Changing the outlook: A strategy for developing and modernising mental health services in prisons* (www.doh.gov.uk/prisonhealth/mhstrategy.pdf)

Department of Health (2003) *Attendances at Accident and Emergency Departments and Minor Injury Units, Strategic Health Authorities in England, 2002–03*. London.

Department of Health (2004). *Choosing Health: Making Healthy Choices Easier.* http://www.dh.gov.uk/PublicationsAndStatistics/Publications/PublicationsPolicyAndGuidance/PublicationsPolicyAndGuidanceArticle/fs/en?CONTENT_ID=4094550&chk=aN5Cor. Accessed 22/4/2005.

Drummond, D.C., Phillips, T., Coulton, S., Barnaby, B., Keating, S., Sabri, R. & Moloney, J. (2003) *Saturday Night and Sunday Morning: the 2003 twenty-four hour national survey of alcohol-related attendances at accident and emergency departments in England*. Final report to the Cabinet Office. September 2003.

Drummond, D.C. (1997) Alcohol interventions: Do the best things come in small packages? *Addiction,* 92, 375-379.

Drummond, D.C. (1990) The relationship between alcohol dependence and alcohol‑related problems in a clinical population. *British Journal of Addiction*, 85, 357‑366.

Drummond, D.C., James. D., Coulton, S., Parrot, S., Baxter, J., Ford, D., Godfrey, C., Lervy, B., Peters, T., Russell, I. & Williams, J. (2003) *The effectiveness and cost effectiveness of screening and stepped care intervention for alcohol use disorders in the primary care setting. Final Report to The Welsh Office for Research and Development.* November 2003.

Drummond, D.C., Oyefeso, N., Phillips, T., Cheeta, S., DeLuca, P., Winfield, H., Jenner, J., Cobain, K., Galea, S., Saunders, V., Perryman, K., Fuller, T., Pappalardo, D., Baker, O. & Christopolous, A. (2005) *National Alcohol Research Project: The 2004 National Needs Assessment for England.* Final Report to the Department of Health. February 2005.

Emmen, M. J., Schippers, G. M., Bleijenberg, G. & Wollsheim, H. (2004). Effectiveness of opportunistic brief interventions for problem drinking in a general hospital setting: systematic review. *British Medical Journal,* 328, 318-322**.**

Fleming, M.F., Mundt, M.O., French, M.T., Manwell, L.B., Stauffacher, E.A. & Barry, K.L. (2002). Brief physician advice for problem drinkers: long-term efficacy and benefit-cost analysis. *Alcoholism: Clinical and Experimental Research,* 26, 36-43.

Flood-Page, C. & Taylor, J.A. (2003). *Crime in England and Wales 2001/02: Supplementary Volume. Home Office Statistical Bulletins 01/03.* London: The Home Office.

Freemantle, N., Gill, P., Godfrey, C., Long, A., Richards, C., Sheldon, T., et al. (1993). Brief Interventions and alcohol use. *Effective Health Care Bulletin No. 7*. University of Leeds: Nuffield Institute for Health.

Friedmann, P.D., McCullough, D., Chin, M.H & Saitz, R. (2000). Screening and intervention for alcohol problems. A national survey of primary care physicians and psychiatrists*. Journal of General Internal Medicine,* 15, 84-91.

Funk, M., Wutzke, S., Kaner, E., Anderson, P., Pas, L., McCormick, R., Gual, A., Barfod, S. (2005) A multi-country controlled trial of strategies to promote dissemination and implementation of brief alcohol intervention in primary health care: Findings of a WHO Collaborative Study. *Journal of Studies on Alcohol*, 66: (in press).

Gentilello, L.M., Rivara, F.P., Donovan, D.M., et al. (1999) Alcohol interventions in a trauma centre as a means of reducing the risk of injury recurrence. *Annals of Surgery,* 230: 473–83.

Green, M., Setchell, J., Hames, P., Stiff, G., Touquet, R. and Priest, R. (1993) Management of alcohol-abusing patients in accident and emergency departments. *Journal of the Royal Society of Medicine* **86**, 393–395.

Green, L.W., Erikson, M.P. and Schor, E.L. (1998) Preventive practices by physicians: Behavioral determinants and potential interventions. *American Journal of Preventive Medicine* **4** (Suppl. No. 4): 101-107.

Heather, N. (1996). The public health and brief interventions for excessive alcohol consumption: the British experience. *Addictive Behaviors*, 21, 857-68.

Heather, N., Dallolio, E., Hutchings, D., Kaner, E. & White, M. (2004). Implementing routine screening and brief alcohol intervention in primary health care: a Delphi survey of expert opinion. *Journal of Substance Use,* 9, 68-85.

Hodgson, R., Alwyn, T., John, B., Thom, B., Smith, A. (2002) The FAST Alcohol Screening Test. *Alcohol & Alcoholism,* 37(1): 61-66.

Hulse, G. K. & Tait, R. J. (2002) Six-month outcomes associated with a brief alcohol intervention for adult in-patients with psychiatric disorders, *Drug & Alcohol Review*, 21 (2), 105 – 112.

Hulse, G. K. & Tait R. J. (2003). Five-year outcomes of a brief alcohol intervention for adult in-patients with psychiatric disorders. *Addiction*, 98, 1061-1068.

Huntly, J.S., Blain, C., Hood, S. & Touquet, R. (2001). Improving detection of alcohol misuse in patients presenting to accident and emergency departments. *Emergency Medicine Journal*, 18, 99-104.

Hutchings, D., Cassidy, P., Kaner, E., Dallolio, E., Pearson, P. & Heather, N. (2005). Qualitative study of patients’ and professionals’ views on implementing alcohol screening and brief interventions in primary care: views from both sides of the desk. *Submitted paper – Primary Care Research and Development.*

Irvin, C. B., Wyer, P. C. & Gerson, L. W. (2000). Preventive care in the emergency department, Part II: Clinical preventive services - an emergency medicine evidence-based review. *Academic Emergency Medicine,* 7, 1042-1054.

Israel, Y., Hollander, O., Sanchez-Craig, M., Booker, S., Miller, V., Gingrich, R. and Rankin, J. G. (1996). Screening for problem drinking and counselling by the primary care physician-nurse team. *Alcoholism: Clinical and Experimental Research,* 20 (8): 1443-50.

Kahan, M., Wilson, L. & Becker, L. (1995). Effectiveness of physician-based interventions with problem drinkers: a review. *Canadian Medical Association Journal,* 152, 851-859.

Kaner, E., Pienaar, E., Campbell, F., Schlesinger, C., Heather, N., Dickinson, H. & Saunders, J. (2003). *Brief interventions for excessive drinkers in primary health care (Protocol)*. Cochrane Library 2003, 2, released 22 April.

Kaner, E., Lock, C., Heather, N., McNamee, P., Bond, S. (2003) Promoting brief alcohol intervention by nurses in primary care: a cluster randomised controlled trial. *Patient Education & Counselling*, 51(3): 277-284.

Kaner, E.F.S., Heather, N., Brodie, J., Lock, C.A., McAvoy, B.R. (2001) Patient and practitioner characteristics predict brief alcohol intervention in primary health care. *British Journal of General Practice*, 51: 822-827.

Kaner, E.F.S., Lock, C.A., McAvoy, B.R., Heather, N., Gilvarry, E. (1999) A randomised controlled trial of training and support strategies to encourage implementation of screening and brief alcohol intervention by general practitioners. *British Journal of General Practice*, 49: 699-703.

Kaner, E. F., Heather, N., McAvoy, B. R., Lock, C. A. & Gilvarry, E. (1999). Intervention for excessive alcohol consumption in primary health care: attitudes and practices of English general practitioners. *Alcohol & Alcoholism,* 34, 559-566.

Kristenson, H., Osterling, A., Nilsson, J. A. & Lindgarde, F. (2002). Prevention of alcohol-related deaths in middle-aged heavy drinkers. *Alcoholism, Clinical and Experimental Research,* 26, 478-484.

Leontaridi, R (2003). *Alcohol Misuse: How Much Does it Cost?* London: Cabinet Office Strategy Unit.

Lock, C.A., Kaner, E.F.S., Heather, N., McAvoy, B.R., Gilvarry, E. (1999) A randomised trial of three marketing strategies to disseminate a screening and brief alcohol intervention programme to general practitioners. *British Journal of General Practice,* 49: 695-698.

Lock C, & Kaner, E. (2000) [Use of marketing to disseminate brief alcohol intervention to general practitioners: promoting health care interventions to health promotors](http://www.ncl.ac.uk/chsr/publications/publication/4563). *Journal of Evaluation in Clinical Practice*, 6(4), 345-357.

Lock, C., Kaner, E., Lamont, S., Bond, S. (2002) A qualitative study of nurses’ attitudes and practices regarding alcohol intervention in primary health care. *Journal of Advanced Nursing,* 39(4): 333-342.

Lock, C. & Kaner, E. (2004) Implementation of brief alcohol interventions by nurses in primary care: do non-clinical factors influence practice? *Family Practice*, 21(3):270-75.

Lock C, Kaner E, Heather N, Purdy S, McNamee P, Doughty J, Crawshaw A, Pearson P. (2005) Effectiveness of nurse-led brief alcohol intervention: A cluster randomised controlled trial. *Journal of Advanced Nursing In press.*

Lockhart, T. (1997) Problem drinkers in Accident and Emergency: health promotion initiatives. *Accident Emergency Nursing*; **5**:16–21

Longabaugh, R., Woolard, R. F., Nirenberg, T. D. *et al.* (2001). Evaluating the effects of a brief motivational intervention for injured drinkers in the emergency department. *Journal of Studies on Alcohol*, 62, 806-816.

Mader, T.J., Smithline, H.A., Nyquist, S. & Letourneau, P. (2001). Social services referral of adolescent trauma patients admitted following alcohol-related injury [*Journal of Substance Abuse Treatment*](http://www.sciencedirect.com/science?_ob=JournalURL&_cdi=5100&_auth=y&_acct=C000047882&_version=1&_urlVersion=0&_userid=912075&md5=ccf6e63199e121107f5562a1471c207a)*,* 21, 167-172

Man et al. (2002) ‘*Dealing with alcohol related detainees in the custody suite’,* Home Office Findings Number 178, Home Office

Mayfield, D., McLeod, G. and Hall, P. (1974) The CAGE questionnaire: validation of a new alcoholism instrument. *American Journal of Psychiatry* **131**, 1121–1123

McAvoy, B.R., Kaner, E.F.S., Haighton, C.A., Heather, N., Gilvarry, E. (1997) Drinkless - marketing a brief intervention package in UK general practice. *Family Practice*, 5: 427-428.

McAvoy, B.R., Kaner, E.F., Lock, C.A., Heather, N., & Gilvarry, E. (1999). Our Healthier Nation: are general practitioners willing and able to deliver? A survey of attitudes to and involvement in health promotion and lifestyle counselling. *British Journal of General Practice*, 49 (440), 181-90.

McCloud, A., Barnaby, B., Omu, N., Drummond, D. C., & Aboud, A. (2004) Relationship between alcohol use disorders and suicidality in a psychiatric population. In-patient prevalence study. *British Journal of Psychiatry,*184, 439-445.

Monteiro, M.G. & Gomel, M. (1998) World Health Organization project on brief interventions for alcohol-related problems in primary health care settings. *Journal of Substance Abuse* **3:** 5-9.

Monti, P.M., Colby, S.M., Barnett, N.P., et al. (1999) Brief intervention for harm reduction with alcohol-positive older adolescents in a hospital emergency department. *Journal of Consulting Clinical Psychology,* 67: 989–94.

MORI (2001). *Report 5: Alcohol and Society.* London: MORI.

Moyer, A., Finney, J., Swearingen, C. & Vergun, P. (2002). Brief Interventions for alcohol problems: a meta-analytic review of controlled investigations in treatment-seeking and non-

treatment seeking populations. *Addiction,* 97, 279-292

medical Research Council (2000) *A framework for development and evaluation of RCTs for complex interventions to improve health.* London: MRC.

National Institute for Clinical Excellence. (2004) *Guide to the Methods of Technology Appraisal*. London: National Institute for Clinical Excellence.

National Primary Care Development Team: Northern NPDT Centre. http://www/ndpt.org/scripts/default.asp?site. Accessed 14/9/2004.

National Primary Care Development Team (2004). The Model For Improvement. http://www.npdt.org/9748/TheModelforImprovement.pdf

Noble A., Best D**.,** Stark MM., Marshall EJ. (2002). *The Role of the Forensic Medical Examiner with “Drunken Detainees” in Police Custody*. Police Research Series Paper 146. Home Office.

Patton, R., Hilton, C., Crawford, M.J., Touquet, R. (2004) The Paddington Alcohol Test: a short report. *Alcohol & Alcoholism*, 39, 266-8.

Patton, R., Crawford, M.J. & Touquet, R. (2004). Hazardous drinkers in the accident and emergency department: who accepts advice? *Emergency Medicine Journal*, 21, 491-492.

Perry, A., Neale, Z., Godfrey, C., McDougall, C., Lunn, J., Glanville, J., Coulton, S. (2005) *The effectiveness of interventions for drug-using offenders in secure establishments and the community*. Centre for Criminal Justice Economics And Psychology, University of York. Report in progress

Peters, J., Brooker, C., McCabe, C. & Short, N. (1998). Problems encountered with opportunistic screening for alcohol-related problems in patients attending an Accident and Emergency department. *Addiction,* 93, 589-594.

Peters, R.H., Greenbaum, P.E., Steinberg, M.L., Carter, C.R., Ortiz, M.M., Fry BC & Valle, S.K. (2000) Effectiveness of screening instruments in detecting substance use disorders among prisoners [*Journal of Substance Abuse Treatment,*](http://www.sciencedirect.com/science?_ob=JournalURL&_cdi=5100&_auth=y&_acct=C000047882&_version=1&_urlVersion=0&_userid=912075&md5=ccf6e63199e121107f5562a1471c207a) [18,](http://www.sciencedirect.com/science?_ob=IssueURL&_tockey=%23TOC%235100%232000%23999819995%23193322%23FLA%23display%23Volume_18,_Issue_4,_Pages_313-418_(June_2000)%23tagged%23Volume%23first%3D18%23Issue%23first%3D4%23Pages%23first%3D313%23last%3D418%23date%23(June_2000)%23&_auth=y&view=c&_acct=C000047882&_version=1&_urlVersion=0&_userid=912075&md5=5cfb3d2a2cfffbbd21fa8c555baff75d) 349-358

Piccinelli, M., Tessari, E., Bortholomasi, M., Piasere, O., Semenzin, M., Garzotto, N. & Tansella, M. (1997) Efficacy of the alcohol use disorders identification test as a screening tool for hazardous alcohol intake and related disorders in primary care. *British Medical Journal* 314: 420-424.

Poikolainen, K. (1999). Effectiveness of brief interventions to reduce alcohol intake in primary health care populations: a meta-analysis. *Preventive Medicine,* 28, 503-509.

Prime Minister’s Strategy Unit (2004). *Alcohol Harm Reduction Strategy for England.* London: Cabinet Office.

Prime Minister’s Strategy Unit (2004). *Interim Analytical Report.* http://www.number10.gov.uk/files/pdf/SU%20interim_report2.pdf. Accessed 14/9/2004.

Project MATCH Research Group (1997) Matching Alcoholism Treatments to Client Heterogeneity: Project MATCH post-treatment drinking outcomes. *Journal of Studies on Alcohol.* 58(1):7-29.

Revolving Doors Agency (2002) *Where do they go?,* Housing, Mental Health and Criminal Justice.

Richmond, R., Heather, N., Wodak, A., Kehoe, L. & Webster, I. (1995). Controlled evaluation of a general practice-based brief intervention for excessive drinking. *Addiction,* 90, 119-132.

Roche, A. M. & Freeman, T. (2004). Brief interventions: good in theory but weak in practice. *Drug & Alcohol Review,* 23, 11-18.

Roche, A. M., Hotham, E. D. & Richmond, R. L. (2002). The general practitioner's role in AOD issues: overcoming individual, professional and systemic barriers. *Drug & Alcohol Review*, 21, 223-230.

Rollnick, S., Mason, P. & Butler, C. (1999). *Health Behaviour Change: A Guide for Practitioners*. Edinburgh: Churchill Livingstone.

Royal College of Physicians (2001)**.** *Alcohol – Can the NHS Afford It? Recommendations for a Coherent Alcohol Strategy for Hospitals*. London: Royal College of Physicians.

Saunders, J. B., Aasland, O. G., Babor, T. F., de la Fuente, J. R. & Grant, M. (1993). Development of the Alcohol Use Disorders Identification Test (AUDIT): WHO Collaborative Project on Early Detection of Persons with Harmful Alcohol Consumption--II. *Addiction,* 88, 791-804.

Selzer, M.L. (1971) The Michigan Alcoholism Screening Test (MAST): The quest for a new diagnostic instrument. *American Journal of Psychiatry*, 127, 1653-1658.

Simmons, J. & Dodd, T. (2003). *Crime in England and Wales 2002/03.* London: Home Office.

Singleton et al. (1997) *Psychiatric morbidity among prisoners*, Office of National Statistics.

Skinner, H.A., Holt, S., Schuller, R., Roy, J. & Israel, Y. (1984) Identification of alcohol abuse using laboratory tests and a history of trauma. *Annals of Internal Medicine*, 101(6):847-51.

Smith, S.G.T., Touquet, R., Wright, C., & Das, D.N (1996) Detection of alcohol misusing patients in accident and emergency departments: the Paddington Alcohol Test (PAT*), Accident & Emergency Medicine,* 13, 308-312

Smith, A. J., Hodgson, R. J., Bridgeman K. & Shepherd, J. P. (2003). A randomised controlled trial of a brief intervention after alcohol-related facial injury. *Addiction*, 98, 43-52.

The EuroQol Group. (1990) "EuroQol - A new Facility for the Measurement of Health-Related Quality of Life". *Health Policy*, 16(3);199-208.

Thom, B., Herring, R. and Judd, A. (1999) Identifying alcohol-related harm in young drinkers: the role of Accident and Emergency departments. *Alcohol and Alcoholism* 34, 910–915.

Wallace, P., Cutler, S. & Haines, A. (1988). Randomized controlled trial of general practitioner intervention with excessive alcohol consumption. *British Medical Journal*, 297, 663-668.

Waller, S., Thom, B., Harris, S. and Kelly, M. (1998) Perceptions of alcohol-related attendancies in accident and emergency departments in England: a national survey. *Alcohol and Alcoholism* **33**, 354–361

Whitlock, E. P., Polen, M. R., Green, C. A., Orleans, T. & Klein, J. (2004). Behavioral counselling interventions in primary care to reduce risky/harmful alcohol use by adults: a summary of the evidence for the US Preventive Services Task Force. *Annals of Internal Medicine,* 140, 557-568.

WHO-CHOICE Cost-effectiveness analyses. WHO European Region A, Addictive substances. <http://www3.who.int/whosis/cea/cea_data.cfm?path=evidence,cea,cea_results&language=english>. Accessed 22/4/2005.

Wilk, A. I., Jensen, N. M. & Havighurst, T. C. (1997). Meta-analysis of randomized control trials addressing brief interventions in heavy alcohol drinkers. *Journal of General Internal Medicine,* 12, 274-283.

Williams, R., Vinson, D.C. (2001) Validation of a single screening question for problem drinking. *Journal of Family Practice*, 50:307-12.

Williams, S., Brown, A., Patton, R., Crawford, M.J. & Touquet, R. (2005). The half-life of the ‘teachable moment’ for alcohol misusing patients in the emergency department. *Drug and Alcohol Dependence*, 77, 205-208.

Williams, B. & Drummond, D.C. (1994) The Alcohol Problems Questionnaire: reliability and validity. *Drug and Alcohol Dependence*, 35, 239-243.

World Advertising Research Centre (2004). *World Drink Trends 2004.* Oxfordshire: Author.

Wright, S., Moran, L. M. M., O'Connor, R. & Touquet, R. (1998). Intervention by an alcohol health worker in an accident and emergency department. *Alcohol & Alcoholism*, 33, 651-656.

Wutzke, S., Conigrave, K., Saunders, J. & Hall, W. (2002). The long-term effectiveness of brief interventions for unsafe alcohol consumption: a 10-year follow-up. *Addiction,* 97, 665-675.
